# Suppression Efficacy of Clubroot on Cruciferous Crops Through Application of the Humic Acid Material

**DOI:** 10.3390/plants14193035

**Published:** 2025-10-01

**Authors:** Shoya Kitabayashi, Fumihiro Nishimura, Takahiro Katayama, Miyu Yoshida, Mitsutaka Mori, Masafumi Saba, Yasuhiro Inoue, Akira Kawaguchi

**Affiliations:** 1 Western Region Agricultural Research Center (WARC) (Kinki, Chugoku and Shikoku Regions), National Agriculture and Food Research Organization (NARO), 6-12-1 Nishifukatsu-cho, Fukuyama 721-8514, Hiroshima, Japan; kitabayashi.shoya332@naro.go.jp; 2 Kagawa Prefecture Agricultural Experiment Station, 1534-1 Ayagawa-cho-kita, Ayauta 761-2306, Kagawa, Japan; xf1540@pref.kagawa.lg.jp (F.N.); td8349@pref.kagawa.lg.jp (T.K.); gr7145@pref.kagawa.lg.jp (M.Y.); js7531@pref.kagawa.lg.jp (M.M.); 3 Institute of Plant Protection, National Agriculture and Food Research Organization (NIPP), 2-1-18 Kannondai, Tsukuba 305-8666, Ibaraki, Japan; saba.masafumi383@naro.go.jp (M.S.); inoue.yasuhiro845@naro.go.jp (Y.I.)

**Keywords:** *Plasmodiophora brassicae*, clubroot, cruciferous crops, humic acid material

## Abstract

Clubroot in cruciferous plants remains a significant threat to growers worldwide. We investigated whether humic acid material (HAM) could control clubroot in Chinese cabbage and broccoli as an alternative to fungicides. Six independent experiments were conducted, and the results were analyzed using a general liner mixed model (GLMM) and a network meta-analysis (NMA). Some HAM treatments significantly controlled clubroot incidence on Chinese cabbage in three experiments, but no significant effects were observed in the others. HAM treatment effects varied across experiments. The GLMM indicated that HAM treatment and the interaction between the HAM amount and planting timing were significantly associated with disease incidence. HAM effectiveness depended on application amount and planting timing after treatment. The NMA estimated a statistically significant risk ratio of 0.85 for planting four weeks after HAM treatment, suggesting low efficacy in suppressing clubroot. Two field trials aligned with the greenhouse results.

## 1. Introduction

Chinese cabbage (*Brassica rapa* L. subsp. *pekinensis*) and broccoli (*Brassica oleracea* L. var. *italica*) are widely cultivated worldwide, including Japan, and is one of the most important vegetables in human diet. However, clubroot disease, which causes root galling in cruciferous plants, has a severe negative impact on the growth of cruciferous crops. Clubroot is a soil-borne disease caused by *Plasmodiophora brassicae* and poses a significant challenge to the cultivation of cruciferous crops, including Chinese cabbage and broccoli. In Japan, growers control clubroot using some chemical fungicides such as amisulbrom and fluazinam, calcareous materials, and planting disease-resistant varieties of cruciferous crops [1]. Nowadays, however, reducing the use of chemicals such as chemical pesticides and chemical synthetic fertilizers has been strongly recommended by countries worldwide. The Ministry of Agriculture, Forestry, and Fisheries [2] in Japan has set 2030 targets under its “MIDORI (green) Strategy for Sustainable Food Systems,” which include a 10.6% reduction in carbon dioxide emissions, a 20% reduction in chemical fertilizer use, and a 10% reduction in chemical pesticide use [2]. In line with this national initiative, it is essential to explore new disease control methods for sustainable and eco-friendly food systems, including organic agriculture. In essence, organic agriculture emphasizes minimizing or eliminating the utilization of synthetic pesticides and fertilizers. Instead, it relies on natural processes and methods to effectively manage pests, diseases, and soil fertility.

Humic acid material (HAM), composed of humic and fulvic acids derived from natural sources, is a type of soil amendment. Researchers have focused on HAM due to its ability to improve soil fertility and health because it plays several important roles, such as enhancing soil physical and biochemical properties by improving structure, texture, water-holding capacity, microbial population, and nutrient availability (especially micronutrients by chelating and co-transporting them to plants) and reducing the mobility of toxic heavy metals by precipitating them [3]. HAM has been reported as one of the plant-biostimulants, promoting nutrient uptake and nutrient use efficiency [4,5]. However, there is few evidence that HAM may inhibit soil-borne diseases, and growers who especially prefer organic farming desire to know the features of HAM for suppressing plant diseases. Consequently, this study seeks to address the existing knowledge gap concerning its impact on soil-borne diseases, particularly clubroot, and to assess its potential efficacy. In this study, we evaluated the effectiveness of HAM in controlling clubroot in Chinese cabbage and broccoli plants.

## 2. Results and Discussion

### 2.1. Regression Analysis by a General Liner Mixed Model (GLMM)

**Figure 1:**
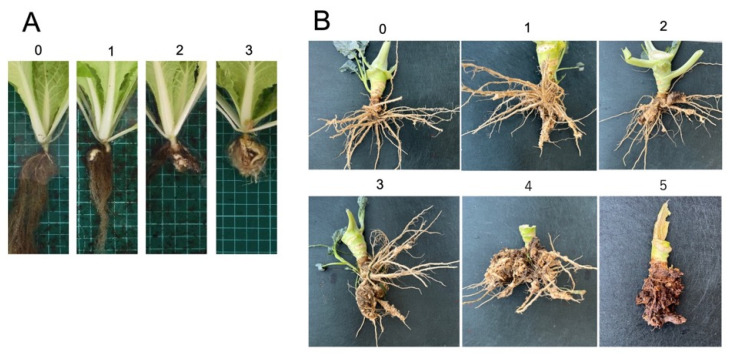
Criterion of clubroot severity (**A**); Clubroot severity of Chinese cabbage plants (greenhouse experiment), clubroot severity using a 0 to 3 scale (0 = no galling, 1 = few small galls (small galls on less than one-half of the roots), 2 = moderate galling (small to medium galls in less than one-half of the roots), 3 = severe galling (medium to large galls in more than on-half of the roots)) (**B**); Clubroot severity of broccoli plants (field experiment), clubroot severity using a 0 to 5 scale (0 = no galling, 1 = few small galls (small galls on less than one-fourth of the roots), 2 = small galling (small to medium galls in less than one-half of the roots), 3 = moderate galling (medium to large galls in more than on-half of the roots)). 4 = severe galling (medium to large galls in more than on-half of the roots), 5 = severe damage and rot (all roots were rotten due to large galls).

**Figure 2:**
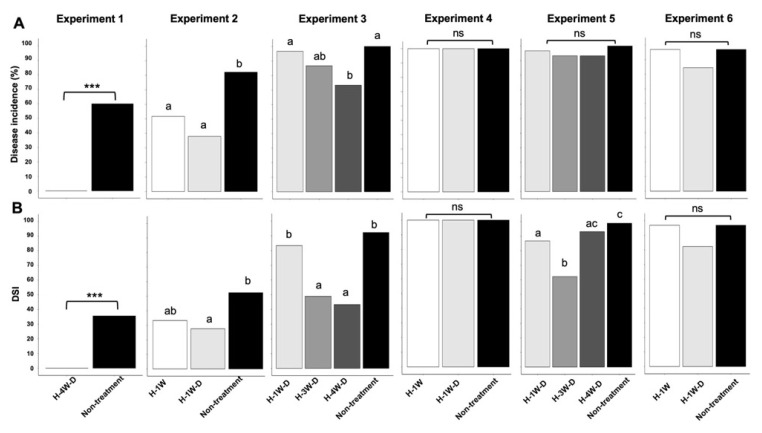
The effectiveness of humic acid material (HAM) in controlling clubroot in Chinese cabbage plants. H-1W; planting at 1 week after treatment with the standard amount of HAM, H-1W-D; planting at 1 week after treatment with double the standard amount of HAM, H-3W-D; planting at 3 weeks after treatment with double the standard amount of HAM, H-4W-D; planting at 4 weeks after treatment with double the standard amount of HAM. (**A**) Disease incidence (%) in six different experiments. In experiment 1, to compare disease incidence between H-4W-D treatment and non-treatment, *p*-value was calculated as 3.4 × 10^−4^ using Fisher’s exact test, with significance levels of *** *p* < 0.001 (*n* = 15). In experiments 2 and 3, different letters represent significant differences according to Ryan’s multiple-comparison test (*p* ≤ 0.05, *n* = 28 to 30). In experiments 4, 5, and 6, no significant differences among treatments according to Ryan’s multiple-comparison test (ns, *p* > 0.05, *n* = 30 to 32). (**B**) Disease severity index (DSI) in six different experiments. In experiment 1, to compare disease incidence between H-4W-D treatment and non-treatment, *p*-value was calculated as 5.8 × 10^−4^ using Wilcoxon rank sum test, with significance levels of *** *p* < 0.001 (*n* = 15). In experiments 2, 3 and 5, different letters represent significant differences according to Wilcoxon rank sum test with Holm’s *p*-value adjustment method (*p* ≤ 0.05, *n* = 28 to 30). In experiments 4 and 6, no significant differences among treatments according to Wilcoxon rank sum test with Holm’s *p*-value adjustment method (ns, *p* > 0.05, *n* = 30 to 32). Equivalent to water treatment was implemented and labeled as non-treatment control.

The effects of HAM treatment varied across the six experiments (Figure 1 and Figure 2A,B, Table 1). While some HAM treatments significantly controlled the disease incidence of clubroot on Chinese cabbage in experiments 1, 2, and 3, no significant effects were observed in experiments 4, 5, and 6 (Figure 2A). In contrast, in the assessment using disease severity index (DSI), some HAM treatments significantly mitigated disease severity of clubroot on Chinese cabbage in experiments 1, 2, 3, and 5 (Figure 2B). A GLMM analysis of disease incidence after HAM treatment showed that the significant (*p* ≤ 0.05) explanatory variables were “HAM treatment” and “Interaction effect between the amount of HAM and planting timing” (Table 2). These results suggest that factors such as the amount of HAM and planting timing play a significant role in the efficacy of HAM treatment in this study (Table 2). The incidence of clubroot caused by *P. brassicae* is known to be strongly influenced by the balance of day- and night-time cycles as well as circadian oscillations in plants [6]. Since pathogens are known to manipulate plant hormones and significantly affect energy homeostasis, it is possible that these processes play a key role in mediating pathogen-induced modifications of the host’s internal clock [7]. However, in the GLMM analysis, explanatory variables such as average day length and sunshine duration were not significant and were excluded from the best-fit model (Table 2), likely because all experiments were conducted in a well-regulated greenhouse maintained at a constant temperature of 23 °C. As a result, disease incidence may not have been strongly influenced by day length or circadian oscillations.

### 2.2. Evaluating the Control Effect of HAM by a Network Meta-Analysis (NMA)

**Figure 3:**
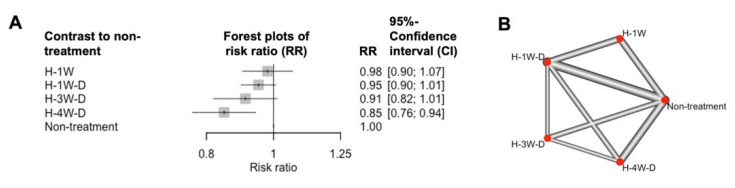
Evaluation based on a network meta-analysis (NMA) of the treatment effects of HAM on clubroot disease in a total of six experiments. (**A**) In the forest plots, each gray square marks the value of the risk ratio compared to the water treatment. The spread (horizontal line) indicates the 95% confidence interval. (**B**) A network map of the meta-analyses. The connected lines show direct comparisons, and the un-connected lines show indirect comparisons. A thicker line indicates a larger number of plants.

An NMA of the six experiments showed that the total estimated RRs of H-1W (planting at 1 week after treatment with the standard amount of HAM), H-1W-D (planting at 1 week after treatment with double the standard amount of HAM), H-3W-D (planting at 3 weeks after treatment with double the standard amount of HAM), and H-4W-D (planting at 4 weeks after treatment with double the standard amount of HAM) compared with non-treatment were 0.98 (95% confidence interval (CI): 0.90–1.07, *p* = 0.637), 0.95 (95% CI: 0.90–0.52, *p* = 0.089), 0.91 (95% CI: 0.82–1.01, *p* = 0.083), and 0.85 (95% CI: 0.76–0.94, *p* = 0.0021), respectively (Figure 3A). In the six experiments, H-1W was indirectly compared to H-3W-D and H-4W-D each other (Figure 3B). The *I*^2^ and *τ*^2^ values were 0.513 and 0.003, respectively. Additionally, the results of the overall *Q* test, the heterogeneity *Q* test (within designs), and the inconsistency *Q* test (between designs) were *p* = 0.020, *p* = 0.004, and *p* = 0.594, respectively. These results indicated the presence of heterogeneity between study designs (*p* ≤ 0.05) and the absence of inconsistency between study designs (*p* > 0.05). The observed heterogeneity would be attributed to the amount of HAM and planting timing, because those factors significantly influenced the RRs, in the result of GLMM (Table 2). Therefore, since two distinct factors—the amount of HAM and planting timing—influenced the RRs, it appeared that the heterogeneity was detected by NMA in this study (Table 2).

The results of the NMA showed that the H-4W-D treatment alone significantly controlled clubroot (Figure 3A). Compared with other treatments, H-4W-D had the longest planting timing among all treatments, and the amount of HAM used was double the standard amount. This result was consistent with the findings of the GLMM analysis (Table 1). For effective clubroot management using HAM treatment, appropriate application conditions, such as the amount of HAM and planting timing, are essential. However, based on the NMA results, the treatment effect of H-4W-D, despite being significant, was limited, as the RR value was 0.85 (RR = 0; disease completely controlled, RR = 1.0; disease not controlled at all).

Interestingly, in experiments 1 and 2, where the disease incidences (%) in each non-treatment group were 60.0 and 82.1, respectively, some HAM treatments significantly and strongly controlled clubroot on Chinese cabbage (Figure 2A, Table 1), suggesting that HAM treatment may be recommended under low-level infection conditions in the soil. Disease severity index (DSI) showed the same trend in experiments 1 and 2, where the DSI in each non-treatment group were 35.6 and 52.4, respectively. HAM treatments showed low DSI values, ranging from 0 to 33.3 in experiments 1 and 2 (Figure 2B, Table 1). In experiment 3, on the other hand, H-3W-D and H-4W-D treatments showed significantly low DSI values 48.9 and 43.3 in spite of DSI value 98.9 in non-treatment (Figure 2B, Table 1). However, the trend of this result was not replicated in experiments 4 and 6 (Table 1), suggesting that HAM treatments could not reduce the number of severely damaged plants with clubroot symptoms under high-level infection conditions in the soil.

### 2.3. Field Trials

We conducted two field trials in gray lowland soil to assess the efficacy of HAM1 and HAM2 treatments. Detailed information on the field experiments is provided in Table 3. In the two field trials conducted, broccoli plants were severely damaged by clubroot. The average disease incidence in each non-treatment plot was exceptionally high, reaching 90.0% in trial ID-1 (Table 3) and 100% in trial ID-2 (Table 3). Despite RR values of 0.93 and 1.00 (HAM2) and 0.99 (HAM1) across two field trials, the average DSI values in the HAM1 and HAM2 treatments were significantly lower compared to those in the non-treatment group (Table 3). These findings corroborate the observed trend in greenhouse experiments (Figure 2 and Figure 3, Table 1), indicating that HAM treatment may be a viable strategy for mitigating the detrimental effects of clubroot on cruciferous crops in commercial settings (Table 3).

Based on the evaluation of DSI values, HAM treatments statistically and significantly reduced the DSI of clubroot. However, the DSI values in HAM treatments remained high (Table 1 and Table 3), suggesting that HAM treatments can mitigate the damage caused by clubroot on cruciferous crops, but they do not have a substantial impact on controlling the spread of clubroot disease. Thus, especially in organic farming, which is strictly limited using chemical fungicides, HAM treatment should not be used alone but combined with other agricultural practices such as applying calcareous materials to adjust soil pH, implementing crop rotation, and/or planting disease-resistant varieties [7,8]. Plus, double dosage of HAM could increase cost. In our future study, we should investigate the control efficacy of a combination of several methods, including HAM, and assess the cost–benefit ratio.

Meanwhile, the mechanisms behind clubroot suppression through HAM treatment remain unknown. It appears that HAM could contribute to clubroot control by raising soil pH [3]. However, in field experiments, the pH did not rise after HAM treatments, remaining around 5.8. To gain further insights for future studies, we employed quantitative PCR (qPCR) to determine whether HAM influences pathogen populations and employed next-generation amplicon sequencing methods to assess whether HAM influences the diversity of soil microorganisms. Furthermore, there is a report suggesting that HAM could influence the dynamics of reactive oxygen species (ROS) in plants, which are signaling molecules crucial for plant development and stress responses [9]. In parallel, HAM has been demonstrated to influence the dynamics of ROS, including superoxide anion and hydrogen peroxide, which play pivotal roles as signaling molecules in plant development [9]. Additionally, we should investigate the gene expression levels associated with ROS response and/or plant disease resistance. To date, however, we have not obtained evidence of alterations in pathogen populations and the diversity of microorganisms as a result of HAM treatments (Supplementary Figure S1). We have a plan to continue investigating the biological mechanism by which HAM suppresses clubroot. Additionally, it is imperative to assess the long-term impact of continuous HAM application over several years on soil microbial communities.

## 3. Materials and Methods

### 3.1. Greenhouse Experiments

Detailed information on six greenhouse experiments (experiment ID 1–6) is provided in Table 1. All experiments were conducted in Fukuyama, Hiroshima, Japan. Resting spores were extracted from fully grown and decaying galls on roots of Chinese cabbage collected from clubroot infected fields in Awaji, Hyogo, Japan. Resting spores were prepared from samples of the gall mixture and stored at −20 °C according to Zhang et al. [10]. To create contaminated soil, resting spore suspensions were introduced as the pathogen by pouring onto commercial soil (Genkikun Kasai 200, containing N = 200 mg/L, P = 3000 mg/L, K = 150 mg/L, pH = 6.5; Katakura Corp Agri, Tokyo, Japan). Specifically, the spore suspension was adjusted to 2 × 10^4^/mL using a haemocytometer. The spore viability was assessed and confirmed by the CFW-PI dual staining method [11]. To inoculate the soil, 50 µL of a spore suspension was added into 1 g of soil (50 µL/g of soil) for soil inoculation, resulting in a final concentration of approximately 10^3^ resting spores/g soil. Here, 60 g of contaminated soil was placed into each plastic pot (11.5 cm in diameter and 10.5 cm in depth). HAM1 (Fujimin, containing humic acids and fulvic acids at 63 g/L, Japan Conservation Engineers, Tokyo, Japan) was diluted 500-fold and poured at a rate of 1.67 mL/pot (27.8 mL/kg soil) as the standard amount according to the protocol of HAM or 3.34 mL/pot (55.6 mL/kg soil) as double the standard amount. Four to six small Chinese cabbage seedlings (cv. Musou, 1-week-old) were planted in each pot at 1, 3, or 4 weeks after HAM1 treatment, and three or six pots per treatment were prepared (Table 1). Equivalent to water treatment was implemented and labeled as non-treatment control (Table 1). Plants were then cultivated in a greenhouse at a constant 23 °C with bottom-watering supply to keep enough soil moisture (around 80%) for infection under natural sunshine conditions, and gall formation on the roots was assessed after four to five weeks.

### 3.2. Regression Analysis

As described above, two different amounts of HAM and three different planting timing were combined as different treatments in this study (Table 1). To elucidate the factors influencing the suppression of clubroot across six greenhouse experiments, a regression analysis based on a GLMM was conducted. The experimental methods adhered to the procedures outlined in previous reports [6,12]. The parameters, including the amount of HAM (categorical numbers: 0 = non-treatment, 1 = standard, 2 = double) and planting timing (categorical numbers: 0 = non-treatment, 1 = 1 week after HAM treatment, 2 = 3 weeks after HAM treatment, 3 = 4 weeks after HAM treatment), were coded and defined as explanatory variables. HAM treatment and the interaction effect between the amount of HAM and planting timing were also incorporated as an explanatory variable, with non-treatment serving as the reference. Average of sunshine duration (h) and average of day-length (h) were also defined as explanatory variables. The data of sunshine duration (h) and day-length (h) in Fukuyama were collected from the National Astronomical Observatory (https://www.nao.ac.jp/en/ (accessed on 25 August 2025)) and the Automated Meteorological Data Acquisition System (AMeDAS) of the Japanese Meteorological Agency (http://www.jma.go.jp/jma/indexe.html (accessed on 25 August 2025)), respectively. The explanatory variable was treated as a fixed effect. Individual experiments (categorical numbers from 1 to 6) were designated as the y-intercepts of random effects. Risk ratio (RR), characterized by the number of diseased plants and the number of healthy plants, was considered the objective variable. RR was defined in this study as RR = (proportion of diseased plants with HAM treatment)/(proportion of diseased plants with non-treatment). The R software (ver. 4.2.2, R Development Core Team) package “lme4” was utilized to estimate regression coefficients.

Then, several models were developed by varying the combinations of explanatory variables. To select the optimal model on a balance between parsimony (limiting the number of parameters to the minimum necessary to explain the data) and goodness of fit, a stepwise selection of the explanatory variables was conducted. Specifically, several models have been developed using all combinations of explanatory variables, and the values of Akaike’s information criterion (AIC) [13] in all models were calculated. The best model, with the lowest value of AIC, was selected (Table 2).

### 3.3. Network Meta-Analysis (NMA)

To combine the findings from independent experiments, disease incidence rates from the six different experiments were subjected to a network meta-analysis (NMA) using a random effects model. NMA has been used to combine direct and indirect evidence on multiple studies comparing multiple treatment [14,15,16]. The experimental methods adhered to the procedures outlined in previous reports [15,16]. NMA based on the frequentist method was carried out using R package “netmeta” (version 2.9-0) [14]. The effect size of the HAM treatment was computed as the total estimated RR. In assessing the treatment effect, a lower RR indicated a higher treatment effect. To assess heterogeneity and inconsistency in the NMA results, Cochran’s *Q* test, *I*^2^, and *τ*^2^ values were calculated by the R package “netmeta”.

### 3.4. Field Experiments

The two field experiments were conducted in an experimental field (gray lowland soil, pH = 5.8 (range = 5.6 to 6.4), soil moisture = around 10%) in Kagawa Prefecture agricultural experiment station, Ayauta, Kagawa, Japan. The two commercial HAM products including HAM1 (Fujimin, the same HAM used in six greenhouse experiments described above) and HAM2 (Keido Premium, containing humic and fulvic acids at 630 g/kg, Life-in, Tokyo, Japan) was used. HAM1 was applied into the soil at a rate of 4.0 L/m^2^ as double the standard amount of the protocol of HAM1. HAM2 was applied into the soil at a rate of 90 g/m^2^ according to the protocol of HAM2 product. After application, HAM products were mixed with soil by plowing, and soil was covered with plastic mulching films. Three or four weeks after application, broccoli seedlings (1 month old) were planted in each plot (2 rows spaced 28 cm per plot). Gall formation on the roots was assessed after three to four months.

### 3.5. Disease Assessment Methods

In greenhouse experiments, the efficacy of HAM1 was evaluated as disease incidence (%) and clubroot severity using a 0 to 3 scale (0 = no galling, 1 = few small galls (small galls on less than one-half of the roots), 2 = moderate galling (small to medium galls in less than one-half of the roots), 3 = severe galling (medium to large galls in more than on-half of the roots)) (Figure 1A). The disease severity index (DSI) was defined as follows:

DSI = 100 × {(1 × *n*_1_) + (2 × *n*_2_) + (3 × *n*_3_)}/(*N* × 3) (1)

where *n*_1_, *n*_2_ and *n*_3_ are the number of diseased plants in each clubroot severity scale (1, 2, and 3) described above, and *N* is the total number of investigated Chinese cabbage plants.

In field experiments, the efficacy of HAM products was evaluated as disease incidence (%) and clubroot severity using a 0 to 5 scale (0 = no galling, 1 = few small galls (small galls on less than one-fourth of the roots), 2 = small galling (small to medium galls in less than one-half of the roots), 3 = moderate galling (medium to large galls in more than on-half of the roots)). 4 = severe galling (medium to large galls in more than on-half of the roots), 5 = severe damage and rot (all roots were rotten due to large galls)) (Figure 1). The disease severity index (DSI) was defined as follows:

DSI = 100 × {(1 × *n*_1_) + (2 × *n*_2_) + (3 × *n*_3_) + (4 × *n*_4_) + (5 × *n*_5_)}/(*N* × 5) (2)

where *n*_1_, *n*_2_, *n*_3_, *n*_4_ and *n*_5_ are the number of diseased plants in each clubroot severity scale (1, 2, 3, 4, and 5) described above, and *N* is the total number of investigated broccoli plants.

## 4. Conclusions

In conclusion, this is the first report unveiling the efficacy of HAM treatment for clubroot disease. Under optimal conditions (double amount of HAM + 4-week planting interval), HAM significantly reduced clubroot incidence by 15% (RR = 0.85, *p* = 0.0021), but its efficacy was weaker than that of chemical fungicides and requires integration with other control measures. To achieve the sufficient effect of HAM treatment, growers must carefully apply it while considering field conditions, including pathogen infection levels and integration with other agricultural practices.

## Figures and Tables

**Table 1:** Results and detail of each greenhouse experiment.

Experiment ID	Treatment ID ^a^	Amount of Humic Acid Material (HAM1) for Pouring onto Soil ^b^	Category of HAM1 Amount	Timing for Planting (Weeks After Treatment)	Category of Timing for Planting	Avarage of Day-Length (h)	Average of Sunshine Duration (h)	No. of Total Plants	No. of Plants per Pot	No. of Pots per Treatment	Disease Incidence (%)	Risk Ratio (RR) ^c^	No. of Plants in Each Disease Severity Scale ^d^				Disease Severity Index (DSI) ^e^
													0	1	2	3	
1	H-4W-D	Double the standard amount	2	4 weeks	3	12.3	5.3	15	5	3	0	0	15	0	0	0	0
	Non-treatment	-	0	-	0			15	5	3	60.0	-	6	5	1	3	35.6
2	H-1W	The standard amount	1	1 week	1	10.5	5.4	29	4 to 5	6	51.7	0.63	14	4	8	3	33.3
	H-1W-D	Double the standard amount	2	1 week	1			29	4 to 5	6	37.9	0.46	18	2	5	4	27.6
	Non-treatment	-	0	-	0			28	4 to 5	6	82.1	-	5	7	11	5	52.4
3	H-1W-D	Double the standard amount	2	1 week	1	11.3	4.7	30	5	6	96.7	0.97	1	2	8	19	83.3
	H-3W-D	Double the standard amount	2	3 weeks	2			30	5	6	86.7	0.87	4	14	6	6	48.9
	H-4W-D	Double the standard amount	2	4 weeks	3			30	5	6	73.3	0.73	8	9	9	4	43.3
	Non-treatment	-	0	-	0			30	5	6	100	-	0	1	5	24	92.2
4	H-1W	The standard amount	1	1 week	1	14.2	4.9	30	5	6	100	1	0	0	0	30	100
	H-1W-D	Double the standard amount	2	1 week	1			30	5	6	100	1	0	0	0	30	100
	Non-treatment	-	0	-	0			30	5	6	100	-	0	0	0	30	100
5	H-1W-D	Double the standard amount	2	1 week	1	14.0	7.9	30	5	6	96.7	0.97	2	0	6	22	86.7
	H-3W-D	Double the standard amount	2	3 weeks	2			30	5	6	93.3	0.93	2	7	14	7	62.2
	H-4W-D	Double the standard amount	2	4 weeks	3			30	5	6	93.3	0.93	1	0	3	25	93.1
	Non-treatment	-	0	-	0			30	5	6	100	-	0	0	1	29	98.9
6	H-1W	The standard amount	1	1 week	1	13.8	9.3	32	5 to 6	6	96.9	1	1	0	0	31	96.9
	H-1W-D	Double the standard amount	2	1 week	1			32	5 to 6	6	84.4	0.87	5	0	2	25	82.3
	Non-treatment	-	0	-	0			32	5 to 6	6	96.9	-	1	0	0	31	96.9

^a^ H-1W; planting at 1 week after treatment with the standard amount of HAM1, H-1W-D; planting at 1 week after treatment with double the standard amount of HAM1, H-3W-D; planting at 3 weeks after treatment with double the standard amount of HAM1, H-4W-D; planting at 4 weeks after treatment with double the standard amount of HAM1. Equivalent to water treatment was implemented and labeled as non-treatment control. ^b^ The standard amount of HAM; 27.8 mL of a 500-fold diluted solution per kg of soil based on the protocol of HAM1. ^c^ RR = (proportion of diseased plants with HAM treatment)/(proportion of diseased plants with non-treatment). ^d^ Clubroot severity of Chinese cabbage plants (greenhouse experiment), clubroot severity using a 0 to 3 scale (0 = no galling, 1 = few small galls (small galls on less than one-half of the roots), 2 = moderate galling (small to medium galls in less than one-half of the roots), 3 = severe galling (medium to large galls in more than on-half of the roots)). ^e^ DSI = 100 × {(1 × *n*_1_) + (2 × *n*_2_) + (3 × *n*_3_)}/(*N* × 3), where *n*_1_, *n*_2_, and *n*_3_ are the number of diseased plants in each clubroot severity scale (1, 2, and 3), and *N* is the total number of investigated Chinese cabbage plants.

**Table 2:** Parameter estimates for the best-fit general linear mixed model (GLMM) for factors related with clubroot of Chinese cabbage in six experiments.

Objective Variable	Explanatory Variable	Parameter Estimate ^b^	Standard Error	*z* Value	*p* Value	Significance ^c^
Risk ratio (RR)	*y*-Intercept ^a^	4.122	1.152	3.579	3.5 × 10^−5^	***
	Humic acid material (HAM) treatment	−1.109	0.538	−2.061	0.039	*
	Interaction effect between the amount of HAM1 and planting timing	−0.371	0.114	−3.260	1.1 × 10^−3^	**

AIC (Akaike’s information criterion) = 39.59. ^a^ Standard error of each experiment, which was defined as the y-intercept of random effects, was estimated as 2.539. ^b^ The estimated regression coefficient is significantly negative, indicating that the explanatory variable negatively impacts the RR value. This suggests that clubroot incidence is effectively suppressed. ^c^ * *p* ≤ 0.05, ** *p* < 0.01, *** *p* < 0.001.

**Table 3:** The result of field experiments for control Clubroot on broccoli using another commercial HAM products.

Trial ID	Planting Date	Rating Date	HAM Product ^a^	Plot ID	Timing for Planting (Weeks After Treatment)	No. of Total Plants	No. of Plants in Each Disease Severity Scale ^b^						Disease Incidence (%)	Risk Ratio (RR) ^c^	*p* Value (Fisher’s Exact Test) ^c^	Disease Severity Index (DSI) ^d^	Wilcoxon Rank Sum Test ^e^
							0	1	2	3	4	5					
1	27 September 2023	16 January 2024	HAM2	1	3 weeks	30	8	4	3	14	1	0	73.3			37.3	
				2	3 weeks	30	4	9	7	8	2	0	86.7			36.7	
				3	3 weeks	30	3	12	3	9	3	0	90.0			38.0	
				Average	-								83.3	0.93	*p* = 0.2728 ^ns^	37.3	*p* = 0.0002 ***
			Non-treatment	1	3 weeks	30	3	5	4	13	5	0	90.0			48.0	
				2	3 weeks	30	6	7	8	8	1	0	80.0			34.0	
				3	3 weeks	30	0	0	0	13	16	1	100			72.0	
				Average	-								90.0	-		51.3	
2	25 March 2025	5 June 2025	HAM1	1	4 weeks	30	0	5	8	12	5	0	100			51.3	
				2	4 weeks	30	0	0	0	12	18	0	100			72.0	
				3	4 weeks	30	1	4	9	16	0	0	96.7			46.7	
				Average	-								98.9	0.99	-	56.7	a
			HAM2	1	4 weeks	30	0	0	0	5	25	0	100			76.7	
				2	4 weeks	30	0	0	0	11	19	0	100			72.7	
				3	4 weeks	30	0	6	7	16	1	0	100			48.0	
				Average	-								100	1.00	-	65.8	b
			Non-treatment	1	4 weeks	30	0	0	1	4	25	0	100			76.0	
				2	4 weeks	30	0	0	0	2	28	0	100			78.7	
				3	4 weeks	30	0	0	0	15	15	0	100			70.0	
				Average	-								100	-		74.9	c

^a^ HAM1; Fujimin, HAM2; Keid Premium. ^b^ Clubroot severity of broccoli plants (field experiment), clubroot severity using a 0 to 5 scale (0 = no galling, 1 = few small galls (small galls on less than one-fourth of the roots), 2 = small galling (small to medium galls in less than one-half of the roots), 3 = moderate galling (medium to large galls in more than on-half of the roots)). 4 = severe galling (medium to large galls in more than on-half of the roots), 5 = severe damage and rot (all roots were rotten due to large galls)). ^c^ RR = (proportion of diseased plants with HAM treatment)/(proportion of diseased plants with non-treatment). ^d^ DSI = 100 × {(1 × *n*_1_) + (2 × *n*_2_) + (3 × *n*_3_) + (4 × *n*_4_) + (5 × *n*_5_)}/(*N* × 5), where *n*_1_, *n*_2_, *n*_3_, *n*_4_ and *n*_5_ are the number of diseased plants in each clubroot severity scale (1, 2, 3, 4, and 5), and *N* is the total number of investigated broccoli plants. ^e ns^ *p* > 0.05, *** *p* < 0.001, different letters represent significant differences according to Wilcoxon rank sum test with Holm’s *p*-value adjustment method (*p* ≤ 0.05).

## Data Availability

The data presented in this study are available upon request from the corresponding author.

## Supplementary Materials

The following supporting information can be downloaded at: https://www.mdpi.com/article/10.3390/plants14193035/s1. Figure S1. The result of principal coordinates analysis (PCoA) summarizing variations in soil fungal communities based on ITS amplicon sequencing data of the field experiment (Trial ID 2 in Table 3).
