# Supplementary material for: Suppression Efficacy of Clubroot on Cruciferous Crops Through Application of the Humic Acid Material
Source: Plants (Basel). 2025 Oct 1;14(19):3035. doi: 10.3390/plants14193035 (PMC12525820; doi:10.3390/plants14193035)
Supplement: Supplementary file 1 [file plants-14-03035-s001.zip › plants-3864375-supplementary.pdf]

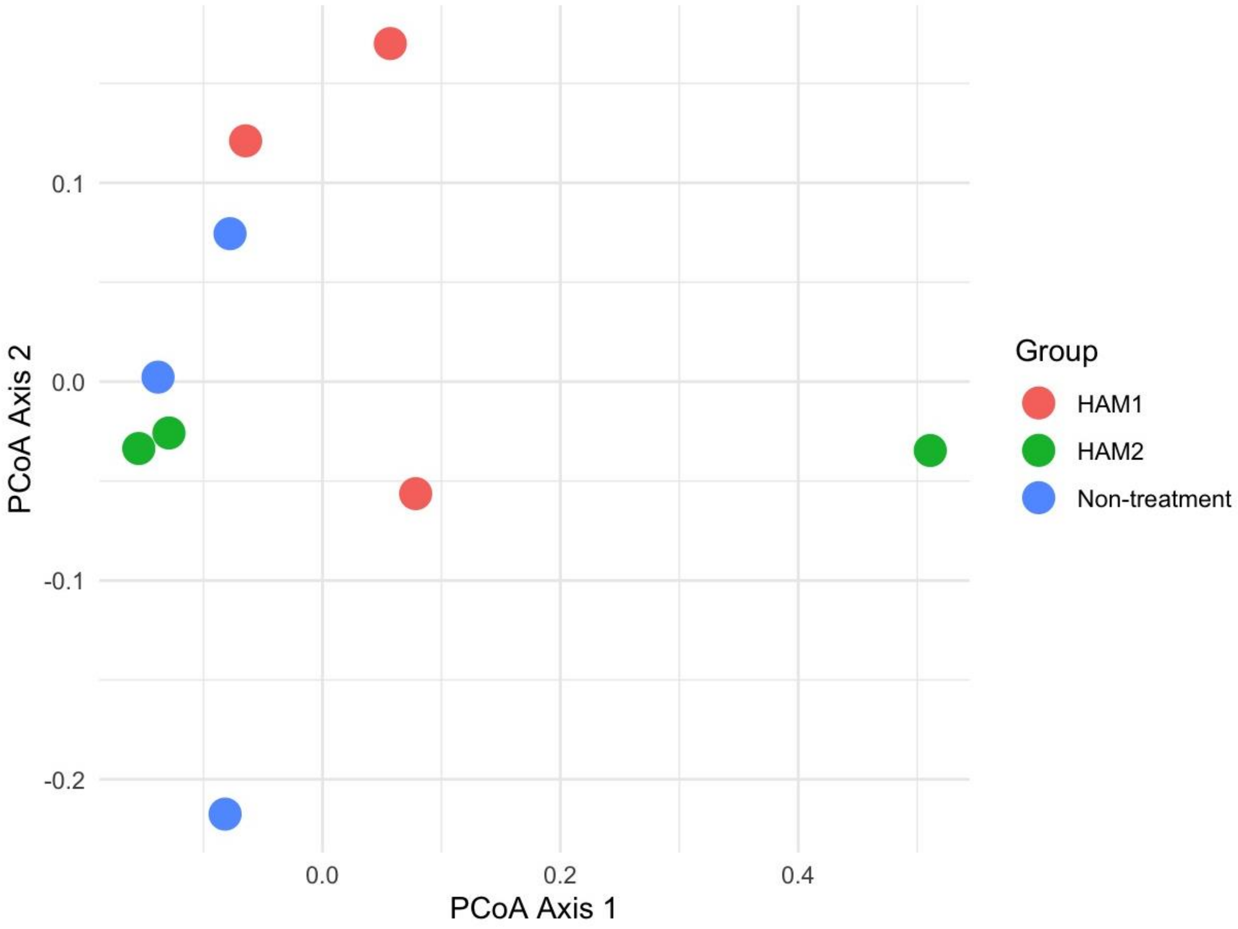

Supplementary Figure S1. The result of principal coordinates analysis (PCoA) summarizing variations in soil fungal communities based on ITS amplicon sequencing data of the field experiment (Trial ID 2 in Table 3). There was no significant difference (adonis: $R^2 = 0.20714$, $p = 0.774$) among each treatment plot (n=3 per treatment).
